# Burkitts lymphoma of the small intestine: A cytological diagnosis

**DOI:** 10.4103/0970-9371.71879

**Published:** 2010-07

**Authors:** Ritesh Sachdev

**Affiliations:** Super Religare Laboratories (Formerly, SRL Ranbaxy Pvt. Ltd.), Clinical Reference Lab, Sector-18, Udyog Vihar, Gurgaon, India

**Figure 1:**
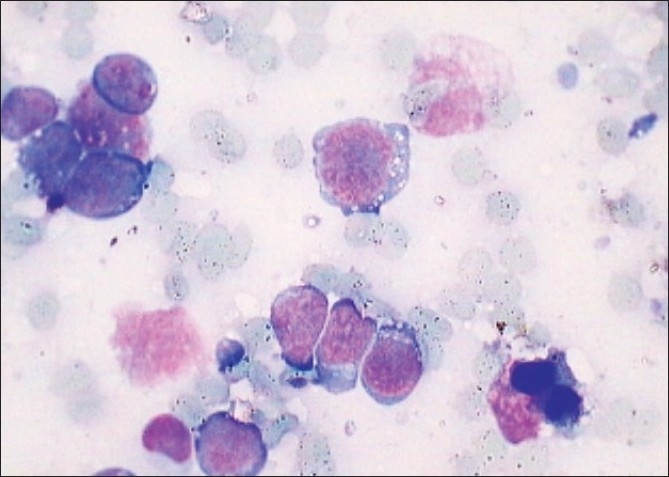
Lymphoma cells with vesicular chromatin and prominent cytoplasmic vacuolization. Background shows lymphoglandular bodies (Giemsa, ×400)

Sir,

We describe a case of Burkitt’s lymphoma (BL) of the small intestine diagnosed on ultrasound-guided fine needle aspiration cytology (FNAC). BL is a high-grade lymphoma usually involving the mandible and the intestines. An early diagnosis is paramount for prompt and effective management. FNAC forms a rapid and effective tool towards an early detection of these lymphomas. A 5-year-old boy presented with abdominal discomfort and progressively increasing abdominal mass. The computed tomography (CT) scan revealed markedly thickened bowel loops with multiple enlarged lymph nodes. There were well-defined hypoechoic nodular lesions in both the kidneys. A suspicion of lymphomatous origin was raised. Ultrasound-guided FNAC was performed through the thickened bowel. Smears were stained with Giemsa and Papanicolaou stains. Many monomorphic, round to oval single cells were identified. These cells displayed a high nucleo-cytoplasmic ratio, vesicular chromatin and prominent cytoplasmic vacuolization [Figure 1]. Many lymphoglandular bodies were seen scattered in the background. A cytological diagnosis of BL of the small intestine was made. The patient was put on a chemotherapeutic regimen, which resulted in the regression of the abdominal mass.

The small intestine is a common site of BL in children and is associated with multisystem lesions. Abdominal mass, bone marrow and central nervous system involvement are poor prognostic markers.[1] Total tumor burden is the principal determinant of prognosis. FNAC, along with other ancillary techniques, provides a rapid and simple tool for early diagnosis and treatment. Ogawa *et al*.[2] used FNAC as a primary tool in the diagnosis of BL of bilateral breasts. Das *et al*.[3] studied 40 cases of BL and found the intra-abdominal location as a common location in Indian patients. FNAC under guidance can help in a rapid diagnosis, thereby assisting in an early treatment in these high-grade lesions.
